# Editorial: New insights into salinity sensing, signaling and adaptation in plants, volume II

**DOI:** 10.3389/fpls.2022.1072658

**Published:** 2022-11-15

**Authors:** Honghong Wu, Camilla Hill

**Affiliations:** ^1^ Ministry of Agriculture (MOA) Key Laboratory of Crop Ecophysiology and Farming System in the Middle Reaches of the Yangtze River, College of Plant Science & Technology, Huazhong Agricultural University, Wuhan, China; ^2^ Hubei Hongshan Laboratory, Chinese Academy of Sciences, Wuhan, China; ^3^ Western Crop Genetics Alliance, Centre for Crop and Food Innovation, Food Futures Institute, Murdoch University, Murdoch, WA, Australia

**Keywords:** nano-biotechnology, nanomaterials, salt sensor, transcription factors, glycine betaine, genome-wide expression

## Plants perceive salt through plant salinity sensors

In the past decade, much progress has been made in identifying the sensors involved in salt-induced stress signaling in plants. Plant roots are the first organs to detect changes in the osmotic potential induced by salt stress. Wang et al. have summarized the recent progress in identifying osmotic and Na^+^ sensors and their signal transduction pathways, specifically in plant roots. Different types of putative sensors in the plasma membrane of root cells exist, including mechanosensory channels (MSCs), which respond to turgor pressure changes of plant cells, plasma membrane-bound protein kinases, which can phosphorylate signaling proteins, and Ca^2+^-mediated ion transporters, such as non-selective cation channels (NSCCs) and cyclic nucleotide-gated channels (CNGCs) which, upon activation, allowing Ca^2+^ to flow into cells. They have further performed bioinformatic analyses of transmembrane domains of receptor-like kinases (RLKs), which are considered as candidate ionic sensors.

## Nano-biotechnological approaches for improving plant salt stress tolerance

Plant nano-biotechnology is an emerging tool to improve plant growth and salt stress tolerance. Nano-enabled plant salt tolerance has been widely reported in many plant species. Li et al. , presented a review paper to illustrate how nanomaterials can be used to improve plant salt tolerance. In this review paper, the mechanisms behind nano-enabled plant tolerance were summarized. It includes maintaining ROS and ion (Na^+^ and K^+^) homeostasis, increasing α-amylase activities, producing gas signaling molecules such as nitric oxide and reducing oxidative damage in membranes *via* decreasing lipoxygenase activities. They highlighted that alleviating oxidative stress damage and maintaining ion homeostasis are commonly employed mechanisms for nano-enabled salt tolerance in plants. Moreover, they also discussed the possible role of phytohormones and the molecular mechanisms in nano-enabled plant salt tolerance.

## Transcription factors involved in salinity stress tolerance in plants

Transcription factors (TFs) play roles in many biological processes, including the regulation of salinity stress tolerance, and have been a target of engineering crops with improved stress tolerance. TFs are well-characterized in the model plant *Arabidopsis* and major crop species such as wheat, but only little is known in pearl millet, a staple food crop grown in the arid and semi-arid regions of Africa and Asia. Awan et al. performed transcriptome profiling of a pearl millet genotype grown under salt stress at three different time points (1, 3, and 7 h after salt treatment). Their study identified TFs mostly upregulated at 1 and 7 h of salt treatment which belonged to different families, including APETALA2/ethylene-responsive element binding factors (AP2-ERF), Basic helix-loop-helix (bHLH), MYB, NAC, and WRKY. Additionally, TFs related to the biosynthesis of phytohormones including abscisic acid (ABA), jasmonic acid (JA), and gibberellic acid (GA) were among the identified differentially expressed genes, and also mostly upregulated at 1 and 7 h of salt treatment. The availability of the transcriptome profiling data sets is a valuable resource to the pearl millet research community and may help identify valuable traits to improve salinity stress tolerance in pearl millet.

Han et al. showed that LbMYB48, an R1-type MYB TF, was strongly induced under salt stress in *Limonium bicolor*, a dicotyledonous recretohalophyte with several multicellular salt glands on the leaves. Salt gland density and salt secretion capacity were impaired in LbMYB48-silenced lines, resulting in reduced salt resistance. RNA-seq analysis showed that LbMYB48 modulates genes related to epidermal cell development, such as *LbCPC-like* and *LbDIS3*, and salt stress-related genes, i.e. *LbSOSs*, *LbRLKs*, and *LbGSTs* to regulate salt gland development and thus salt tolerance in *Limonium bicolor*. Further, heterologous over-expression of *LbMYB48* improved salt tolerance in *Arabidopsis thaliana*. The authors conclude that *LbMYB48* regulates the salt gland development and salt tolerance by regulating the expression of epidermal cell development-related genes in *Limonium bicolor*.

## Maintaining Na^+^ homeostasis is important for plant salt tolerance

Over-accumulation of Na^+^ is toxic to most plants. Zhu et al. investigated the mechanisms underlying glycine betaine-improved maize salt tolerance *via* the maintenance of Na^+^ homeostasis. Non-invasive micro-test technology (NMT) and confocal microscope imaging showed that glycine betaine application canincrease leaf and root Na^+^ efflux and alleviate cytosolic Na^+^ over-accumulation in maize under salt stress. Glycine betaine significantly upregulated the expression of plasma membrane H^+^-ATPase genes *ZmMHA2*, and *ZmMHA4* as well as Na^+^/H^+^ antiporter gene *ZmNHX1*, and also improved vacuolar activity of *NHX*. However, no significant effect of glycine betaine on V type H^+^-ATPases was detected. Taken together, their results showed that glycine betaine modulated PM H^+^-ATPase to help to maintain cellular Na^+^ homeostasis, ultimately showing improved salt tolerance in maize.

Salt Overly Sensitive 1 (SOS1) is a well-characterized Na^+^ efflux transporter, but only limited knowledge exists of its role in soybean salt stress responses. To understand *SOS1* gene function in soybean, Zhang et al. created three *gmsos1* mutants using the CRISPR-Cas9 gene editing system. *gmsos1* mutants were hypersensitive to salt stress and accumulated higher root Na^+^ levels but much lower levels of root K^+^ under salt stress compared to the wild-type. The transcriptomic profiles of the *gmsos1* mutant roots showed that many differentially expressed genes encode proteins with functions in ion transport and response to abiotic stress. In summary, the authors provide evidence that *SOS1* function is conserved across eudicots such as *Arabidopsis* and soybean, as well as monocots such as rice.

## Author contributions

All authors listed have made a substantial, direct and intellectual contribution to the work, and approved it for publication.

## Funding

This work was supported by the NSFC grant (No. 32071971, 31901464), Hubei Agricultural Science and Technology Innovation Center Program (2021-620-000-001-032), project 2662020ZKPY001 supported by the Fundamental Research Funds for the Central Universities, and joint project SZYJY2021008 from Huazhong Agricultural University and Agricultural Genomics Institute at Shenzhen, Chinese Academy of Agricultural Sciences to HW.

## Conflict of interest

The authors declare that the research was conducted in the absence of any commercial or financial relationships that could be construed as a potential conflict of interest.

## Publisher’s note

All claims expressed in this article are solely those of the authors and do not necessarily represent those of their affiliated organizations, or those of the publisher, the editors and the reviewers. Any product that may be evaluated in this article, or claim that may be made by its manufacturer, is not guaranteed or endorsed by the publisher.

